# Reference values for methacholine reactivity (SAPALDIA study)

**DOI:** 10.1186/1465-9921-6-131

**Published:** 2005-11-04

**Authors:** Pierre-Yves Jayet, Christian Schindler, Nino Künzli, Jean-Pierre Zellweger, Otto Brändli, André Paul Perruchoud, Roland Keller, Joel Schwartz, Ursula Ackermann-Liebrich, Philippe Leuenberger, SAPALDIA team

**Affiliations:** 1 Service of Pulmonology, University Hospital Lausanne, Switzerland; 2 Institute of Social and Preventive Medicine, University of Basle, Switzerland; 3 Division of Environmental Health, University of Southern California, USA; 4 Zürcher Höhenklinik Wald, Switzerland; 5 Department of Internal Medicine, University Hospital of Basle, Switzerland; 6 Klinik Barmelweid, Aarau, Switzerland; 7 Department of Environmental Health, Harvard School of Public Health, USA

## Abstract

**Background:**

The distribution of airway responsiveness in a general population of non-smokers without respiratory symptoms has not been established, limiting its use in clinical and epidemiological practice. We derived reference equations depending on individual characteristics (i.e., sex, age, baseline lung function) for relevant percentiles of the methacholine two-point dose-response slope.

**Methods:**

In a reference sample of 1567 adults of the SAPALDIA cross-sectional survey (1991), defined by excluding subjects with respiratory conditions, responsiveness during methacholine challenge was quantified by calculating the two-point dose-response slope (O'Connor). Weighted L1-regression was used to estimate reference equations for the 95^th ^, 90^th ^, 75^th ^and 50^th ^percentiles of the two-point slope.

**Results:**

Reference equations for the 95^th ^, 90^th ^, 75^th ^and 50^th ^percentiles of the two-point slope were estimated using a model of the form a + b* Age + c* FEV_1 _+ d* (FEV_1_)^2 ^, where FEV_1 _corresponds to the pre-test (or baseline) level of FEV_1_. For the central half of the FEV_1 _distribution, we used a quadratic model to describe the dependence of methacholine slope on baseline FEV_1_. For the first and last quartiles of FEV_1_, a linear relation with FEV_1 _was assumed (i.e., d was set to 0). Sex was not a predictor term in this model. A negative linear association with slope was found for age. We provide an Excel file allowing calculation of the percentile of methacholine slope of a subject after introducing age – pre-test FEV_1 _– and results of methacholine challenge of the subject.

**Conclusion:**

The present study provides equations for four relevant percentiles of methacholine two-point slope depending on age and baseline FEV_1 _as basic predictors in an adult reference population of non-obstructive and non-atopic persons. These equations may help clinicians and epidemiologists to better characterize individual or population airway responsiveness.

## Background

Description of normal airway responsiveness in a general population is a recent concept [1]. However its use in clinic or in epidemiological studies is limited by the lack of established norms (as percentiles) of the distribution of airway reactivity [2] according to the age, sex and airway caliber of the subjects [3].

The conventional method to measure bronchial responsiveness is to perform a bronchochallenge test where FEV_1 _is measured at increasing levels of methacholine [4] up to a maximal dose of 2 mg and to evaluate the resulting dose-response curve. Results of the test are usually expressed by an index of responsiveness, the provocating dose (PD20) or concentration (PC20) producing a 20% fall of FEV_1_. A subject is defined to be hyperreactive if, at any of the methacholine levels tested, his/her FEV_1 _falls below 80% of the baseline value. In epidemiological studies, however, the concept of hyperreactivity has substantial limitations since the majority of subjects do not reach the critical threshold level so that their degree of responsiveness cannot be defined in terms of a critical dose [5].

In order to obtain a simple index of non-specific airway reactivity for every subject (hyperreactive or normal), O'Connor et al [6] defined the slope of the dose-response curve as the ratio between percent decline of FEV_1 _(from the post-saline value to the value measured after the final methacholine dose administered) and the final cumulative dose of methacholine. For both asthmatic and normal people this simple dose-response slope provides a good summary of each subject's dose-response curve [7].

The distribution of hyperreactivity or of airway responsiveness in a general population sample has been described in several studies [6,8,9]. For tests performed with methacholine or with histamine, non-specific airway responsiveness shows a unimodal skewed distribution. Although asthmatic subjects tend to lie in the "reactive" tail of the distribution, there is a considerable overlap between the distributions of asthmatic and non-asthmatic subjects. Some authors suggest that this unimodal distribution reflects several overlapping clinical states between normal subjects and symptomatic asthmatics [10]. However, apart from clinical state many individual predictive factors influence the degree of bronchial responsiveness. Whereas age has been investigated in many studies [2,5,11-16], the exact influence of aging on reactivity is still not clear. Its estimated effect appears to depend on whether other possible confounding variables such as baseline lung function or smoking status are simultaneously taken into account. Sex appears to be another important predictive factor: women seem to be more reactive than men [5,12,14-16], but adjusting for possible confounding factors may explain some of this difference. Pre-test FEV_1 _is considered as a major parameter influencing bronchial responsiveness [11,12,14-16]. However many other potential variables appear to play a role, such as smoking status [11,13-15,17], geographic characteristics [2,11], atopic status [14-16], occupational exposure to inhalation irritants [18], presence of chronic respiratory conditions or prior asthma [19], or recent upper airway infection [19]. These findings indicate that bronchial responsiveness, as described by PD20, PC20 or dose-response slope, may be influenced by a wide range of factors that in turn, may substantially affect its interpretation.

Data from the asymptomatic never smoking participants of the SAPALDIA cross-sectional study (1991) have already been used by Brändli [20,21] to derive reference equations for mean values and lower limits of normal of spirometric lung function. In this paper we use data of the methacholine challenge test from a selected sample of "normal" participants of the SAPALDIA sample to establish reference equations for some important percentiles of methacholine slope depending on important individual characteristics (i.e., sex, age and baseline lung function).

## Methods

SAPALDIA (Swiss Study on Air Pollution and Lung Diseases in Adults) is a multicenter study designed to investigate the relationship between exposure to air pollutants and respiratory symptoms or diseases. The eight study areas participating in the project were chosen to represent the variety of environmental conditions found in Switzerland concerning geography, climate, degree of urbanisation and air pollution. The study was approved by the institutional review board for human investigations of the different areas. In the cross-sectional part performed in 1991, a random sample of adults 18 to 60 years old were invited to take part in the study. 9651 subjects were included in the study, representing 59% of all eligible subjects. Health assessment included a detailed questionnaire, computer-based spirometric tests, methacholine bronchial challenge and skin allergy tests to 8 inhalative allergens. Details on the methodology of these assessments are given elsewhere [22].

Spirometry measurements were done using a Sensor-Medics 2200 pulmonary function system SP (Bilthoven, The Netherlands). This is an open sensor device which meets the quality criteria of the American Thoracic Society. The Sensor-Medics spirometer displays an error code after each forced expiration to inform the technician about the acceptability of the maneuver and the reproducibility between the trials using the standard quality criteria defined by the American Thoracic Society [23]. The trials were recorded electronically on a personal computer as they were done. Calibration was done at least once daily, using a 3-liter syringe. All the spirometry technicians were trained together according to a standardized protocol and were tested on volunteers [24]. Each of the following criteria was sufficient for excluding a subject from the methacholine test: a) a baseline FEV_1 _/ FVC ratio of less than 80% of the ECCS-norm [25], b) a baseline FEV_1 _of less than 70% of the ECCS-norm, c) pregnancy or breast feeding, d) a myocardial infarction within the three months preceding the SAPALDIA examination, e) severe heart failure under treatment, f) treatment with β-blockers including eye-drops, g) refusal to participate. These exclusions and the requirement of having complete and valid data on lung function and bronchial responsiveness reduced the sample size to 6942. Non-specific bronchial reactivity was tested using methacholine chloride (Provocholine^® ^, Roche, Nutley, New Jersey, USA) prepared in 0.39, 1.56, 6.25, and 25.0 mg/ml solutions in a phosphate buffer without phenol. Increasing concentrations of methacholine were administered through an aerosol dosimeter (Mefar MB3, Bovezzo, Italy) up to a cumulative dose of 2 mg (8.37 μmol). With each inhalation, approximately 0.01 ml was delivered to the subject. The first dose inhaled by the subject was a saline control. The schedule was then 4 inhalations of methacholine of 0.39 mg/ml (total dose 0.016 mg), 3 inhalations of 1.56 mg/ml (cumulative dose 0.062 mg), 3 inhalations of 6.25 mg/ml (cumulative dose 0.25 mg), 3 inhalations of 25 mg/ml (cumulative dose 1 mg), and 4 inhalations of 25.0 mg/ml (total cumulative dose 2 mg). If a decrease in FEV_1 _of more than 10% from the baseline level occurred at any intermediate point of the test, smaller increments (i.e., halving the doses and doubling the number of inhalations) were introduced. Testing continued until the final dose of 2 mg was administered or until FEV_1 _had fallen by 20% or more. Under this protocol the cumulative doses of methacholine converted in micromoles at each level were 0, 0.065, 0.26, 1.05, 4.18, and 8.37. At each level, the subjects were asked to inhale slowly from their functional residual capacity up to their vital capacity. The subjects were instructed to keep a full inspiration for 4 seconds before a slow normal exhalation. After each dose level of methacholine, 2 forced expiratory maneuvers were performed at 1 and 2 minutes after the end of the methacholine inhalation and the best of the two FEV_1 _values was considered [26].

**Figure 1:**
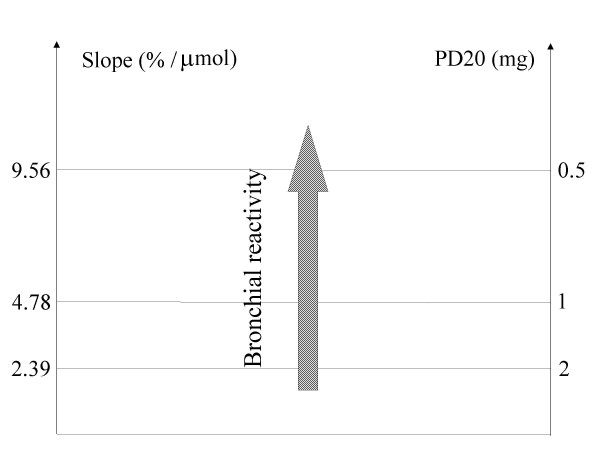
**Graphic representation of the relationship between the two-point dose response slope and PD20**. This figure shows the relationship between the two-point dose response slope and PD20. The horizontal line drawn at a slope of 2.39% decrease/μmol represents the "cut-off" threshold commonly used to define bronchial hyperreactivity (20% decrease of FEV_1 _after a cumulative methacholine dose of ≤ 2 mg).

Methacholine responsiveness was quantified by calculating the two-point dose-response slope as defined by O'Connor [6]. Slope is defined as the percentage of decline of FEV_1 _from the post-saline value to the value measured after the final methacholine dose administered divided by the final cumulative methacholine dose administered. Figure 1 provides a schematic diagram illustrating the relationship between the two-point dose response slope (expressed in % decline of FEV_1 _divided by the final cumulative methacholine dose administered) and PD20 (provocating dose in mg producing a 20% fall of FEV_1_). The figure demonstrates that higher reactivity is indicated by a higher value of slope. The horizontal line drawn at a slope of 2.39% decrease/μmol represents the threshold commonly used to define bronchial hyperreactivity (20% decrease of FEV_1 _after a cumulative methacholine dose of ≤ 2 mg).

Of the participants who performed the methacholine test, only 1567 were included in the reference sample after applying the following exclusion criteria: a) *current or former smoking*: (i.e., having smoked 20 or more packs of cigarettes or more than 360 g of tobacco); b) *a prior diagnosis of asthma *or report of *symptoms related to asthma or bronchitis *(i.e., wheezing in the last 12 months and/or shortness of breath at rest in the last 12 months and/or nocturnal attacks of shortness of breath in the last 12 months and/or attacks of asthma in the last 12 months and/or current asthma medication and/or cough or phlegm on most days of at least three months of the year); c) *atopy*: defined by the presence of at least one positive reaction to the eight inhalant allergens tested in a skin prick test (subjects with missing results in this test were also excluded); d) *recent respiratory infection *(i.e., anamnesis of a respiratory infection within three weeks prior to the methacholine test).

Weighted L1-regression was used to estimate percentile functions. This method consists of finding the model parameters which minimize a given weighted sum of absolute residual values. For instance, estimating the model for the 75^th ^percentile is achieved by assigning the absolute values of positive residuals three times the weight of the absolute values of negative residuals. In general, if the m-th percentile is to be estimated, absolute values of positive residuals are given a weight proportional to 1/(100-m) and absolute values of negative residuals a weight proportional to 1/m. Details of this method are described elsewhere [27-29]. To test whether a given model could be improved by adding an additional predictor term, we defined a dichotomous variable U taking the value 1 for observations with methacholine slopes exceeding the respective percentile estimates and the value 0 for all other observations. A logistic regression model incorporating the covariate part of the underlying percentile model along with the additional predictor term was then computed. If the additional predictor term was significant then it was added to the percentile model. These methods have already been applied in a similar context to estimate percentile equations for lung function [21].

We tested the performance of this approach in identifying asthmatics using the 90^th ^percentile of slope as threshold in subjects who answered positively to the double question: "Have you ever had asthma? Was this confirmed by a doctor?" and performed methacholine test (i.e. fulfilled initial inclusion criteria mentioned above). For both men and women of this subsample, the percentage of subjects whose slopes exceeded this threshold was compared to the percentage of subjects usually defined as hyperreactive (i.e., with a positive response to the methacholine test based on a fall of 20% of FEV_1 _during the test).

## Results

The different stages leading to the selection of the reference sample are described in Table 1. Only 1567 persons, representing 20.9% of all participants of the methacholine bronchial challenge fulfilled all criteria. The major part of subjects excluded were current or former smokers.

**Table 1:** Definition of the study sample, SAPALDIA cross-sectional study, 1991

	Men	Women	Total
			
Whole SAPALDIA sample	4743 (100%)	4908 (100%)	9651 (100%)
- subjects with incomplete data on lung function and bronchial responsiveness*	3446 (72.7%)	3496 (71.2%)	6942 (71.9%)
- current or former smokers	1278 (26.9%)	1770 (36.1%)	3048 (31.6%)
- subjects with a prior diagnosis of asthma or symptoms related to asthma or bronchitis	1052 (22.2%)	1428 (29.1%)	2480 (25.7%)
- subjects with a positive or missing skin test	733 (15.5%)	1107 (22.6%)	1840 (19.1%)
- subjects with recent respiratory infection	612 (12.9%)	955 (19.5%)	1567 (16.2%)
Total of the study sample	612 (12.9%)	955 (19.5%)	1567 (16.2%)

* exclusion criteria from methacholine testing were FEV1/FVC ratio less than 80% of the ECCS-norm, FEV1 of less than 70% of the ECCS-norm, incomplete data on lung function, pregnancy or breast feeding, a myocardial infarction within the 3 months preceding the examination, being treated for severe heart failure, being treated with β-blockers including eye-drops, or refusal to participate; subjects with incomplete data on methacholine test were also excluded.

**Figure 2:**
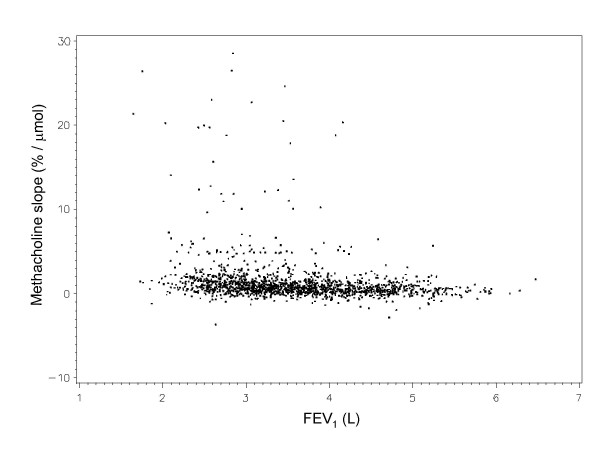
Scatter plot of methacholine slope vs. pretest level of FEV_1 _for our study sample (n = 1567) (excluding 5 observations with slopes >30%/μmol)

Characteristics of the study population are provided in Table 2. It included a higher proportion of women (60.9%) than in the whole methacholine test sample (49.4%), explained by their lower prevalence of current or former smoking. A scatter plot of methacholine slope vs. baseline FEV_1 _(all subjects) is given in Figure 2.

**Table 2:** Distribution of basic predictor variables in the reference sample, SAPALDIA cross-sectional study, 1991

	Men (n = 612)	Women (n = 955)	Entire reference sample (n = 1567)
			
<30 yrs	31.7%	20.8%	25.1%
30–40 yrs	24.8%	20.5%	22.2%
40–50 yrs	25.5%	27.9%	26.9%
≥50 yrs	18.0%	30.8%	25.8%
Height, mean (SD)	176.1 (6.7)	163.5 (6.5)	168.4 (9.0)
Weight, mean (SD)	75.2 (10.2)	61.6 (10.6)	66.9 (12.4)
FEV_1_, mean (SD)	4.33 (0.67)	3.10 (0.54)	3.58 (0.84)
PD20 prevalence*	4.4%	14.6%	10.6%

* PD20 prevalence denotes prevalence of subjects with a fall of 20% or more in FEV_1 _during the methacholine test

**Figure 3:**
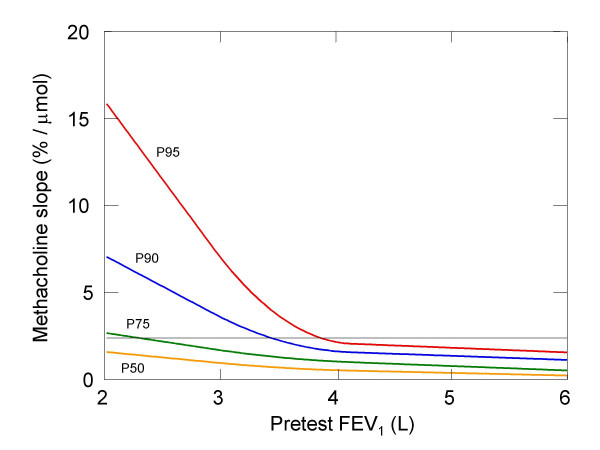
**Percentiles of methacholine slope as a function of pretest level of FEV_1 _(among persons of reference sample aged 40 years)**. This figure shows the percentiles of methacholine slope as a function of pretest level of FEV_1 _(among persons of reference sample aged 40 years). The horizontal line defines the threshold between "hyperreactive" and "normal" subjects as defined by a 20% fall of FEV_1 _from the baseline value before or at the maximal methacholine dose. The scale of pretest level of FEV_1 _extends from below the 1 st percentile to above the 99 th percentile of pretest level of FEV_1 _in our reference sample.

Prediction equations of 95^th ^, 90^th ^, 75^th ^and 50^th ^percentiles of the two-point slope are given in Table 3. The corresponding curves for 40 years old subjects are represented in Figure 3. Prediction equations were derived involving age and pre-test (or baseline) FEV_1_. Between the lower and upper quartile of FEV_1_, these models are of the form: a + b* Age + c* FEV_1 _+ d* FEV_1_^2 ^, whereas no quadratic term in FEV_1 _is used below the 1^st ^and above the 3^rd ^quartile. We thus used natural quadratic splines with knots at the lower and upper quartiles of FEV_1 _to describe the dependency of percentiles of methacholine slope on baseline FEV_1_. Therefore, up to the first quartile of FEV_1_, each percentile curve of slope for a given age is described by a straight line. Another straight line describes the percentile curve for FEV_1_-values above the upper quartile. These two straight line segments are connected by a parabola segment in such a way that the transition between the different pieces is smooth. Although the coefficients a and c have to vary between the three intervals, the smoothness requirement imposes linear restrictions on them. On the other hand, the coefficient b has the same value everywhere, since the association between slope and age appeared to be approximately linear for all percentiles considered. Consequently, the curves for figure 3 would have to be shifted downward and upward for ages higher and lower than 40 years, respectively. The model shows that, with lower pre-test values of FEV_1_, level and spread of the percentiles increases. A horizontal line drawn at y= 2.39% decrease/μmol represents the threshold commonly used to define bronchial hyperreactivity (20% decrease of FEV_1 _after a cumulative methacholine dose of ≤ 8.37 μmol). A higher proportion of subjects belong to this "hyperreactive" category at lower values of FEV_1 _or lower values of age. Consequently a higher proportion of women are defined as "hyperreactive" (Table 3). We provide an additional Excel file allowing calculation of the percentile of methacholine slope of a subject after introducing his/her age, pre-test FEV_1_, and results of methacholine challenge (i.e. methacholine total cumulative dose and percentage of FEV_1 _decline at this total cumulative dose) (Additional file 1).

**Table 3:** Percentiles of methacholine slope* among men and women of the reference sample, SAPALDIA cross-sectional study, 1991

	minimum	P5	P10	P25	P50	P75	P90	P95	maximum
									
men (n = 612)	-2.81	-0.55	-0.25	0.13	0.48	0.98	1.60	2.25	40.5
women (n = 955)	-3.69	-0.13	0.06	0.41	0.90	1.67	3.25	5.72	78.5
entire reference sample (n = 1567)	-3.69	-0.30	-0.07	0.26	0.72	1.41	2.40	4.85	78.5

* final %decrease in FEV_1 _from baseline divided by highest dose of methacholine administered

Among subjects with physician-diagnosed asthma (n = 411), the percentage of subjects with a fall of 20% or more during the methacholine test was significantly higher in women than in men (58.8% vs. 43.8%, p < 0.01). In the same population, percentages of subjects above the 90^th ^percentile of methacholine slope from the model including FEV_1 _did not differ between both sexes (51.0% vs. 51.2%, p = 0.98).

## Discussion

Previous studies have demonstrated that non-specific bronchial responsiveness to methacholine may be influenced by a number of factors [2,5,11-19]. On the basis of a review of the literature we excluded subjects presenting characteristics that may influence bronchial reactivity in a "non-physiological" way from our study population: smokers and former smokers, anamnestic asthmatic or bronchitic subjects, atopics, and persons who reported a recent respiratory infection. Moreover, the methacholine challenge was not performed in subjects with spirometric evidence of airway obstruction. Our preliminary analysis showed that among the potential predictor variables considered (i.e., sex, age, height, weight, FEV_1_, FVC, FEV_1 _/ FVC, FEF_25–75%_, FEF_25–75% _/ FVC), sex, age and either FEV_1_, FEF_25–75%_, or FEF_25–75% _/ FVC had the strongest explanatory power (results not shown). Using pre-test FEV_1 _in addition to basic variables (sex, age, and height) improves prediction equations for methacholine reactivity, probably due to multiple factors. In subjects with restrictive syndrome, whatever the etiology, airway calibre is better described by absolute values of FEV_1 _than by the height or weight of subjects. Moreover, the underlying mechanisms of bronchial responsiveness to a pharmacological agent are complex and multifactorial. Several studies suggested that, apart from lung size, other important determinants of non specific bronchial hyperresponsiveness are airway geometry and properties of smooth muscles. Wassmer [15] showed in an adult German population that BHR (defined by a fall in 10% or 20% of FEV_1 _in methacholine challenge) or bronchial responsiveness (described by dose-response slope) is most strongly predicted by lung function parameters. In a study analyzing hyperreactivity in a large random adult population, Britton [16] showed that FEV_1_, FEV_1 _%predicted and FEV_1 _/ FVC were strongly and independently related to BHR, identifying with varying degrees of overlap separate groups of individuals at increased risk of hyperreactivity. In our analysis, however, FEV_1 _/ FVC was not significantly associated with methacholine slope. This may be explained by the exclusion of obstructive and atopic subjects.

An independent significant effect of age on bronchial methacholine dose-response slope is seen in our population study even after correction for FEV_1_, showing a negative cross-sectional association between slope and age after adjustment for differences in FEV_1_. This is an interesting result per se, given that an independent effect of age on BHR has not been consistently documented in the literature [2,5,11-13,16].

Our percentile equations may be used in epidemiological studies to define more valid individual measures of responsiveness (i.e. severity) because they incorporate inherent confounding factors such as age and pre-test airway calibre. Moreover, the equations may enable clinicians to assess the degree of bronchial responsiveness in their patients with greater validity. We provide a simple Excel file enabling the computation of the percentile of a subject's bronchial responsiveness provided that this value lies between the 50^th ^and the 95^th ^percentile of the distribution in our adult reference population.

In clinical practice, methacholine challenge is currently used primarily to exclude asthma in atypical situations, being recognized as a useful but imprecise test. Using the 90^th ^percentile as a "cut-off" level for identifying asthmatics in our sample of subjects with self-reported physician diagnosed asthma provided a sensitivity of 51.1% which did not differ between sexes; this percentage was very similar to the percentage of subjects with a fall of 20% or more during the methacholine test in the same population (50.9%), where a significant difference was, however, present between sexes (58.8% in women vs. 43.8% in men). We therefore hypothesize that our equations and index provide a more valid individual marker of the clinical severity, enabling better characterization and quantification of bronchial responsiveness. While receiver operator characteristic (ROC) studies would be needed to evaluate the best "cut-off" percentile for asthma diagnosis, using the 90 th percentile yielded the same sensitivity in our subsample of asthmatics as the PD20 criterion in a similar study population of subjects with self-reported physician diagnosed asthma [30].

## Conclusion

The present study provides equations for four relevant percentiles of methacholine slope (defined according to O'Connor) depending on the age and baseline FEV_1 _in an adult reference population of non-obstructive and non-atopic persons. In addition to the fact that such models may help to better understand the underlying mechanisms of BHR, they may be of use in future epidemiological studies to better identify subjects whose bronchial hyperreactivity is caused by extrinsic factors or by obstructive or atopic conditions. It may be of interest to both clinicians and epidemiologists that the sensitivity of our method in identifying subjects with a doctor's diagnosis of asthma is the same in men and women whereas the traditional method based on PD20 has a lower sensitivity in men. More generally, our equations may help physicians to better characterize and follow bronchial responsiveness of individual patients, based on simple predictive factors.

## Competing interests

The author(s) declare that they have no competing interests.

## Authors' contributions

PYJ, CS and PL conducted the analyses and drafted the article. CS, NK, JPZ, OB, APP, RK, JS, UAL and PL contributed to the design of the study, the acquisition of data and the interpretation of data. All authors contributed to the conception of the research question, made important intellectual contributions during the drafting process and have given approval for the final version.

**Table 4:** Estimated equations of the 95^th ^, 90^th ^, 75^th ^and 50^th ^percentiles of methacholine slope given age and pretest level of FEV_1 _(litres), SAPALDIA cross-sectional study, 1991

Slope_95_	=	34.70	-	0.0167	age	-	9.001	FEV_1_	+	0	FEV_1_^2 ^	(FEV_1_≤2.93)
	=	65.69	-	0.0167	age	-	30.152	FEV_1_	+	3.6095	FEV_1_^2 ^	(2.93<FEV_1_≤4.14)
	=	3.82	-	0.0167	age	-	0.266	FEV_1_	+	0	FEV_1_^2 ^	(FEV_1_>4.14)
Slope_90_	=	14.81	-	0.0160	age	-	3.523	FEV_1_	+	0	FEV_1_^2 ^	(FEV_1_≤2.93)
	=	26.48	-	0.0160	age	-	11.483	FEV_1_	+	1.3584	FEV_1_^2 ^	(2.93<FEV_1_≤4.14)
	=	3.19	-	0.0160	age	-	0.236	FEV_1_	+	0	FEV_1_^2 ^	(FEV_1_>4.14)

Slope_75_	=	4.90	-	0.0056	age	-	0.997	FEV_1_	+	0	FEV_1_^2 ^	(FEV_1_≤2.93)
	=	7.53	-	0.0056	age	-	2.796	FEV_1_	+	0.3071	FEV_1_^2 ^	(2.93<FEV_1_≤4.14)
	=	2.27	-	0.0056	age	-	0.253	FEV_1_	+	0	FEV_1_^2 ^	(FEV_1_>4.14)

Slope_50_	=	3.03	-	0.0039	age	-	0.642	FEV_1_	+	0	FEV_1_^2 ^	(FEV_1_≤2.93)
	=	4.77	-	0.0039	age	-	1.828	FEV_1_	+	0.2025	FEV_1_^2 ^	(2.93<FEV_1_≤4.14)
	=	1.30	-	0.0039	age	-	0.152	FEV_1_	+	0	FEV_1_^2 ^	(FEV_1_>4.14)

## Supplementary Material

Additional File 1 **Calculation of percentiles of methacholine slope as a function of pre-test FEV_1 _and age. **This additional Excel file allows calculation of the percentile of methacholine slope of a subject after introducing his/her age, pre-test FEV_1_, and results of methacholine challenge (i.e. methacholine total cumulative dose and percentage of FEV_1 _decline at this total cumulative dose). Click here for file
